# Comparison of the PI-RADS 2.1 scoring system to PI-RADS 2.0: Impact on diagnostic accuracy and inter-reader agreement

**DOI:** 10.1371/journal.pone.0239975

**Published:** 2020-10-05

**Authors:** Andreas M. Hötker, Christian Blüthgen, Niels J. Rupp, Aurelia F. Schneider, Daniel Eberli, Olivio F. Donati

**Affiliations:** 1 Institute of Diagnostic and Interventional Radiology, University Hospital Zurich, Zurich, Switzerland; 2 Department of Pathology and Molecular Pathology, University Hospital Zurich, Zurich, Switzerland; 3 Department of Urology, University Hospital Zurich, Zurich, Switzerland; University of Sydney, AUSTRALIA

## Abstract

**Purpose:**

To assess the value of the PI-RADS 2.1 scoring system in the detection of prostate cancer on multiparametric MRI in comparison to the standard PI-RADS 2.0 system and to assess its inter-reader variability.

**Materials and methods:**

This IRB-approved study included 229 patients undergoing multiparametric prostate MRI prior to MRI-guided TRUS-based biopsy, which were retrospectively recruited from our prospectively maintained institutional database. Two readers with high (reader 1, 6 years) and low (reader 2, 2 years) level of expertise identified the lesion with the highest PI-RADS score for both version 2.0 and 2.1 for each patient. Inter-reader agreement was estimated, and diagnostic accuracy analysis was performed.

**Results:**

Inter-reader agreement on PI-RADS scores was fair for both version 2.0 (kappa: 0.57) and 2.1 (kappa: 0.51). Detection rates for prostate cancer (PCa) and clinically significant prostate cancer (csPCa) were almost identical for both PI-RADS versions and higher for the more experienced reader (AUC, Reader 1: PCa, 0.881–0.887, csPCa, 0.874–0.879; Reader 2: PCa, 0.765, csPCa, 0.746–0.747; both p > 0.05), both when using a PI-RADS score of ≥ 4 and ≥3 as indicators for positivity for cancer.

**Conclusions:**

The new PI-RADS 2.1 scoring system showed comparable diagnostic performance and inter-reader variability compared to version 2.0. The introduced changes in the version 2.1 seem only to take effect in a very small number of patients.

## Introduction

Multiparametric prostate MRI is now part of the standard clinical work-up for patients with elevated PSA at many institutions, as it has shown to improve detection rates of clinically-significant prostate cancer in patients who subsequently undergo targeted biopsy, with fewer biopsy cores necessary [1–6]. In clinical routine, the PI-RADS version 2.0 scoring system [7] has been the most common approach to identifying and scoring suspicious lesions, as it offers much-needed standardization of reports, a structured way of assessing lesions and has been broadly validated [8, 9].

Recently, an updated version of the PI-RADS guidelines, version 2.1, has been published [10], which addresses various inconsistencies and issues that have been identified in studies and by the increased experience over the years of use [11]. In addition to clarification of technical aspects, the revised guidelines induce subtle changes to the scoring of indeterminate lesions in the transitional zone (TZ) and an update to the scoring of lesions on diffusion-weighted sequences (DWI), seeking to reduce the number of lesions scored “indeterminate” and thus further increase diagnostic accuracy of prostate MRI. This is of particular importance, as indeterminate lesions pose a clinical challenge regarding patient management and further course of action (e.g. whether a biopsy is required or not in these patients). A higher precision of the PI-RADS 2.1 guidelines could therefore lead to a reduction in unnecessary biopsies, however, missing clinically significant cancers that could affect patient outcome needs to be avoided.

The purpose of this retrospective analysis was therefore to assess the value of the new PI-RADS 2.1 scoring system in the detection of prostate cancer and to compare it to version 2.0.

## Materials and methods

### Patients and reference standard

This study was approved by the institutional review board (Cantonal Ethics Commission Zurich) and the requirement for a study-specific informed consent for this study was waived. A retrospective search was performed on our prospectively maintained institutional database from 01/2015–12/2017 for consecutive patients undergoing multiparametric prostate MRI following transperineal template saturation biopsy. This initial search yielded a number of 267 patients. Of those, patients who had not signed a general consent to share their data for any research question/ who had withdrawn consent to participate in the study (n = 27) or whose scans demonstrated severe motion or susceptibility artifacts (n = 11) were excluded. The final patient cohort therefore consisted of 229 men (mean age: 63.1, range: 46–79 years), with a mean PSA of 8.2 μg/L (range: 0.81–100 μg/L). The mean time between MRI and biopsy was 42.3 days (0–208 days). All clinical information was collected from our hospital information system. Pre-biopsy PSA values were not available in 4 patients.

Transperineal template saturation biopsy served as the reference standard and was carried-out by board-certified urologists. Cores were taken every 5 mm throughout the prostate up to a total of 40 cores. If a lesion suspicious for tumor (PI-RADS score ≥ 3) had been identified on prior mpMRI, three additional targeted biopsies were taken from this area. All histopathological specimens were evaluated by dedicated genitourinary pathologists. Clinically significant prostate cancer was defined as a Gleason score of ≥ 3 + 4.

Of note, the patients included in this study have been part of earlier investigations [12], however, these studies did not investigate the value of the PI-RADS scoring system version 2.1.

### MRI and image analysis

All MRI scans were acquired on scanners manufactured by Siemens (Siemens Skyra, Siemens Healthineers, Erlangen, Germany) at a field strength of 3 Tesla and using an 18-channel phased-array receiver coil. In 68 patients, an additional balloon-covered expandable endorectal coil (Medrad, Warrendale, USA) was used. The MRI protocol consisted of T2- weighted turbo spin-echo sequences covering the prostate and the seminal vesicles (transverse, sagittal and coronal orientation) and a transverse diffusion-weighted sequence with three b-values (100, 600 and 1000 s/mm^2^). A high b-value of 1400 s/mm^2^ was calculated. Dynamic contrast-enhanced MR images were obtained in transverse orientation with a temporal resolution ≤ 8s. Gadoterate meglumine (Dotarem, Guerbet, Darmstadt, Germany) was used as a contrast agent in a dose of 0.1 mmol/kg of body weight. The MR protocol was in accordance to the general recommendations published in the PI-RADS guidelines [7].

Two readers, a board-certified radiologist (initials blinded for review) with > 5 years of experience in prostate MRI and a radiology resident with 2 years of experience (initials blinded for review) separately reviewed all scans while being blinded to all clinical and histopathological information. Each reader identified the lesion with the highest PI-RADS score on a per-patient basis for the PI-RADS 2.0 [7] and 2.1 [10] scoring system individually. No wash-out period was introduced between PIRADS 2.1 and 2.0 readings to not introduce intra-reader variability as a potential bias.

### Statistical analysis

All statistical analyses were performed in SPSS (IBM Inc., Armonk, USA) and R version 2.13 (The R Foundation for Statistical Computing). Continuous variables were expressed as medians and ranges. Categorical variables were expressed as counts and percentages. Inter- and intra-reader agreement was assessed using weighted Cohen’s kappa and was interpreted as follows: excellent agreement > 0.75, good agreement 0.59–0.75, fair agreement 0.40–0.58, poor agreement < 0.4. Diagnostic accuracy was assessed by the area under the curve of a receiver-operator-characteristics (ROC) analysis for both the detection of prostate cancer and clinically significant prostate cancer (defined as prostate cancer with a highest Gleason score ≥ 3 + 4). ROC curves were compared according to the methodology laid out by DeLong et al. to test for statistical significance [13]. A test result with a p-value < 0.05 was considered statistically significant.

Analyses were performed both with using a PI-RADS score of ≥ 4 and ≥ 3 to indicate positivity for cancer.

## Results

### Patient and tumor characteristics

The number of patients with highest Gleason scores found on histopathological examinations of the biopsy cores were as follows: 26 with 3+3 (11.4%), 68 with 3+4 (29.7%), 31 with 4+3 (13.5%), 11 with 4+4 (4.8%), 10 with 4+5 (4.4%), 1 with 5+4 (0.4%) and one patient with 5+5 (0.4%).

### Inter-reader agreement

Inter-reader agreement between reader 1 and 2 for PI-RADS 2.0 scores was found to be fair (kappa: 0.57, 0.49–0.66 95% CI) and slightly higher than the agreement between reader 1 and 2 on PI-RADS 2.1 scores (kappa: 0.51, 0.44–0.59 95% CI).

### Detection of prostate cancer and clinically significant prostate cancer

**Fig 1:**
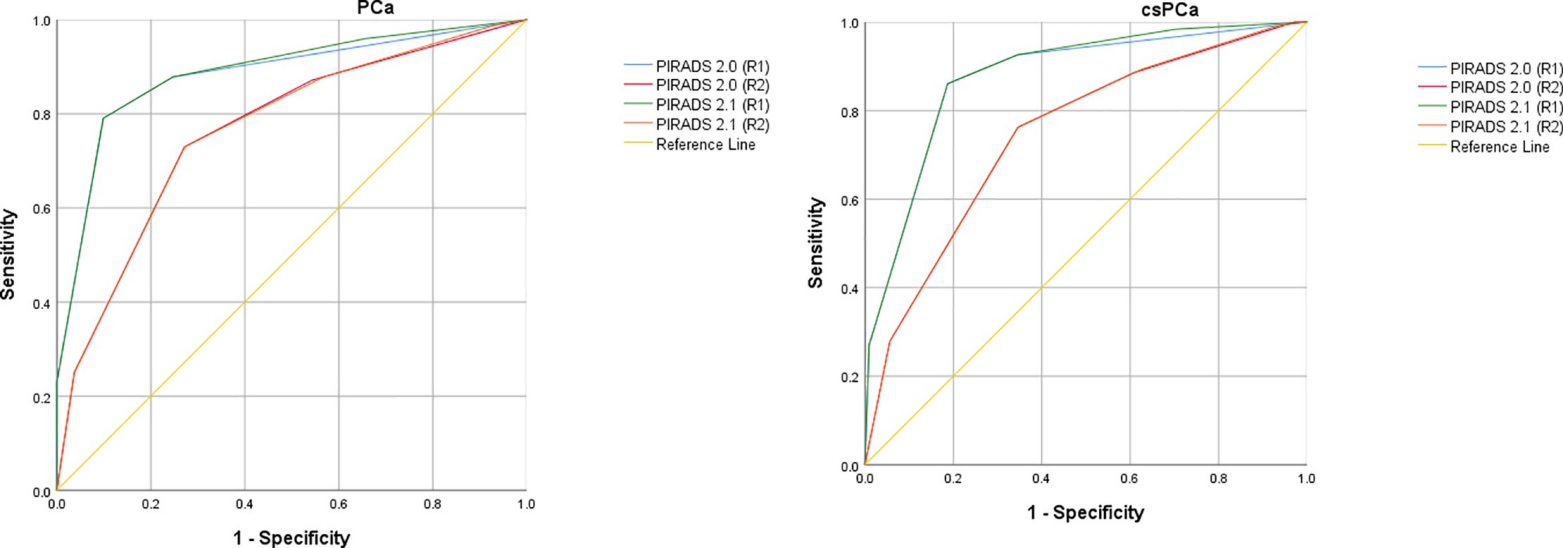


Detailed information on the distribution of PI-RADS scores for both version 2.0 and 2.1 as well as the detected prostate cancers (PCa) or clinically significant prostate cancers (csPCa) and associated sensitivity/specificity are given in Tables 1 and 2, Fig 1 (**Fig 1A:** Receiver-operator-characteristics (ROC) analysis for the detection of prostate cancer with PI-RADS version 2.0 and 2.1 for both readers, respectively. **Fig 1B:** Receiver-operator-characteristics (ROC) analysis for the detection of clinically significant prostate cancer (Gleason score ≥ 3 + 4) with PI-RADS version 2.0 and 2.1 for both readers, respectively.).

**Table 1:** PI-RADS scores version 2.0 and 2.1 for both readers and the number of detected prostate cancers (PCa) and clinically significant prostate cancers (csPCa).

		Reader 1			Reader 2		
PI-RADS 2.0		N (%)	# PCa (%)	# csPCa (%)	N (%)	# PCa (%)	# csPCa (%)
	1	0 (0%)	0/0 (0%)	0/0 (0%)	3 (1.31%)	1/3 (33.33%)	0/3 (0%)
	2	79 (34.50%)	18/79 (22.78%)	9/79 (11.39%)	53 (23.14%)	18/53 (33.96%)	14/53 (26.42%)
	3	25 (10.92%)	13/25 (52.00%)	8/25 (32.00%)	43 (18.78%)	21/43 (48.84%)	15/43 (34.88%)
	4	91 (39.74%)	83/91 (91.21%)	72/91 (79.12%)	90 (39.30%)	71/90 (78.89%)	59/90 (65.56%)
	5	34 (14.85%)	34/34 (100%)	33/34 (97.06%)	40 (17.47%)	37/40 (92.50%)	34/40 (85.00%)
PI-RADS 2.1	1	34 (14.85%)	6/34 (17.65%)	2/34 (5.88%)	8 (3.49%)	2/8 (25%)	1/8 (12.50%)
	2	45 (19.65%)	12/45 (26.67%)	7/45 (15.56%)	45 (19.65%)	16/45 (35.56%)	12/45 (26.67%)
	3	25 (10.92%)	13/25 (52.00%)	8/25 (32.00%)	46 (20.09%)	22/46 (47.83%)	16/46 (34.78%)
	4	91 (39.74%)	83/91 (91.21%)	72/91 (79.12%)	90 (39.30%)	71/90 (78.89%)	59/90 (65.56%)
	5	34 (14.85%)	34/34 (100%)	33/34 (97.06%)	40 (17.47%)	37/40 (92.50%)	34/40 (85.00%)

**Table 2:** Sensitivity and Specificity of PI-RADS 2.0 and PI-RADS 2.1 scores for both readers in the detection of prostate cancer and clinically significant prostate cancer (Gleason score ≥ 3+4). A PI-RADS score ≥ 4 was deemed to indicate positivity for cancer.

				Sensitivity (95% CI)	Specificity (95% CI)
PI-RADS 4+5	Reader 1	Cancer	PI-RADS 2.0	0.791 (0.718–0.848)	0.901 (0.817–0.949)
			PI-RADS 2.1	0.791 (0.718–0.848)	0.901 (0.817–0.949)
		Clinically significant cancer	PI-RADS 2.0	0.813 (0.729–0.876)	0.861 (0.788–0.911)
			PI-RADS 2.1	0.813 (0.729–0.876)	0.861 (0.788–0.911)
	Reader 2	Cancer	PI-RADS 2.0	0.728 (0.623–0.813)	0.730 (0.653–0.795)
			PI-RADS 2.1	0.728 (0.623–0.813)	0.730 (0.653–0.795)
		Clinically significant cancer	PI-RADS 2.0	0.654 (0.560–0.738)	0.762 (0.679–0.829)
			PI-RADS 2.1	0.654 (0.560–0.738)	0.762 (0.679–0.829)
PI-RADS 3+4+5	Reader 1	Cancer	PI-RADS 2.0	0.878 (0.816–0.922)	0.753 (0.649–0.834)
			PI-RADS 2.1	0.878 (0.816–0.922)	0.753 (0.649–0.834)
		Clinically significant cancer	PI-RADS 2.0	0.926 (0.866–0.961)	0.654 (0.560–0.738)
			PI-RADS 2.1	0.926 (0.866–0.961)	0.654 (0.560–0.738)
	Reader 2	Cancer	PI-RADS 2.0	0.872 (0.808–0.916)	0.457 (0.353–0.565)
			PI-RADS 2.1	0.878 (0.816–0.922)	0.432 (0.330–0.541)
		Clinically significant cancer	PI-RADS 2.0	0.885 (0.817–0.930)	0.393 (0.305–0.487)
			PI-RADS 2.1	0.893 (0.826–0.937)	0.374 (0.288–0.468)

An almost identical performance of the PI-RADS 2.1 scoring system compared to the version 2.0 was seen for two different thresholds for indicating positivity for prostate cancer (PI-RADS score of 4–5 or 3–5). AUCs were marginally higher in PI-RADS 2.1 (PCa: reader 1: 0.887, reader 2: 0.765; csPCa: reader 1: 0.879, reader 2: 0.747) compared to 2.0 for both readers (PCa: reader 1: 0.881, reader 2: 0.765; csPCa: reader 1: 0.874, reader 2: 0.746, see Table 2), but the difference between PIRADS 2.1 and 2.0 was not statistically significant for either reader (PCa: reader 1: p = 0.34, reader 2: p = 0.86; csPCa: reader 1: p = 0.17, reader 2: p = 0.82). A lesion demonstrating imaging features which are newly described in the recent update of PI-RADS (e.g. marked hypointensity on ADC/hyperintensity on high b-value DWI but not both or a lesion with a TZ score of 2 and a DWI score of ≥ 4) which would vindicate a higher overall score was not seen in our study.

## Discussion

Multiparametric prostate MRI is part of the clinical pathway of patients with elevated PSA in many centers, as its value in the detection and classification of prostate cancer is supported by a large body of evidence [1, 2, 4, 12, 14]. Despite some minor limitations and inconsistencies becoming apparent after implementation, the PI-RADS 2.0 scoring system has been broadly adopted in the radiological and urological communities and has been extensively validated to allow for the reliable identification of csPCa [8].

The recently published PI-RADS 2.1 guidelines [10] try to remedy some of the limitations identified [11], for example, by clarifying technical aspects of prostate MRI, the reporting of central zone lesions or lesions arising from the anterior fibromuscular stroma. However, the new guidelines also introduce subtle changes to the scoring of both transitional zone tumors and lesions on DWI in general, which is hoped to improve the system’s accuracy and reliability. The criteria for DWI scores 2 and 3 have been revised, with a score of 2 being assigned to lesions that are “linear/wedge-shaped hypointense on ADC and/or linear/wedge-shaped hyperintense on high b-value DWI” whereas a score of 3 requires “focal hypointense on ADC and/or focal hyperintense on high b-value DWI” and a lesion may be “markedly hypointense on high b-value DWI or markedly hyperintense on high b-value DWI, but not both”. In TZ tumors, a lesion with a newly defined T2 score of 2 and a DWI score of 4 or higher would now be assigned an overall score of 3 (instead of 2). However, lesions fulfilling these criteria seem to be rare in clinical routine and we did not see any in our study cohort: Both readers scored lesions nearly identical when using PI-RADS version 2.0 and 2.1 and the small increase in AUC seen in both readers is probably not clinically relevant. Research data on the comparison between PIRADS 2.0 and 2.1 is still sparse, with a few report indicating a slight improvement in the detection of cancer in the transitional zone [15, 16], however, a recent study of Moreira et al. aligns with our results and did not see “significant changes in the number of positive and negative MRI results” and “expected low influence in clinical management” [17]. We did see an effect of reader experience, with the experienced reader reaching higher levels of sensitivity/specificity than the unexperienced reader, even when using the same PI-RADS criteria for the scoring of lesions. This indicates that even when descriptive terms are defined more precisely, the interpretation of these terms remain subjective to a certain extent and are interpreted differently among radiologists. A possible means to further reducing different interpretations of defined descriptions of imaging features may be to introduce quantitative measures.

Another aim of the new guidelines is the “improvement of inter-reader variability”, as reproducibility of findings/scores represents a crucial requirement for any scoring system in clinical routine. However, we did not see an increase in agreement between the two readers when moving from 2.0 to 2.1, albeit a small decrease which is most likely not clinically significant. This decrease may be due to the readers being less familiar with the new scoring system compared to PI-RADS 2.0, however, we could not demonstrate an improvement regarding inter-reader agreement/reproducibility.

The recent changes introduced to the PI-RADS scoring system [10] certainly clarify certain technical matters or aspects of reporting and may help in scoring of few non-typical lesions, but their influence on the majority of “typical” suspicious lesion encountered in clinical routine seems to be small. Nevertheless, the performance of PI-RADS in the detection of clinically significant cancer is good and improves with experience, which highlights the importance of training and structured education in prostate MRI [18]. For further improvement on the detection rates, the use of quantitative imaging parameters may be an option [5, 19, 20].

Our study has limitations: First, we scored one lesion per patient (the “index lesion”) and the results may differ when scoring every lesion in a patient, as this reduces the number of indeterminate lesions (if another lesion with a higher score is present)–though clinical management is most commonly based on the Gleason score of the dominant lesion. Ideally, the use of pathological maps would allow for direct radiological-pathological correlation in future studies. Secondly, this study was limited by its retrospective design and albeit including a relatively high number of patients, may still be limited by the size of the patient cohort since the changes introduced in PI-RADS 2.1 only affect a very small number of lesions.

In conclusion, we demonstrated a comparable performance of PI-RADS 2.1 compared to version 2.0 in the detection of prostate cancer and clinically significant prostate cancer and could not show an improvement in inter-reader agreement. Future revision of the PI-RADS guideline may need to take quantitative measurements into account in order to increase reproducibility of PI-RADS scores.

## Supporting information

S1 Data (XLSX)
